# Neuropsychiatric Systemic Lupus Erythematosus: Molecules Involved in Its Imunopathogenesis, Clinical Features, and Treatment

**DOI:** 10.3390/molecules29040747

**Published:** 2024-02-06

**Authors:** Angel A. Justiz-Vaillant, Darren Gopaul, Sachin Soodeen, Rodolfo Arozarena-Fundora, Odette Arozarena Barbosa, Chandrashehkar Unakal, Reinand Thompson, Bijay Pandit, Srikanth Umakanthan, Patrick E. Akpaka

**Affiliations:** 1 Department of Para-Clinical Sciences, University of the West Indies, St. Augustine Campus, St. Augustine 00000, Trinidad and Tobago; sachin.soodeen@my.uwi.edu (S.S.); chandrashehkar.unakal@sta.uwi.edu (C.U.); reinand.thompson@sta.uwi.edu (R.T.); bijay.pandit@sta.uwi.edu (B.P.); patrick.akpaka@sta.uwi.edu (P.E.A.); 2 Port of Spain General Hospital, University of the West Indies, St. Augustine Campus, St. Augustine 00000, Trinidad and Tobago; darren.gopaul2@my.uwi.edu; 3 Eric Williams Medical Sciences Complex, North Central Regional Health Authority, Champs Fleurs, San Juan 00000, Trinidad and Tobago; rodolfo.fundora@sta.uwi.edu (R.A.-F.); odearobar@gmail.com (O.A.B.); 4 Department of Clinical and Surgical Sciences, Faculty of Medical Sciences, The University of the West Indies, St. Augustine 00000, Trinidad and Tobago

**Keywords:** neuropsychiatric systemic lupus erythematosus, autoantibodies, cytokines, steroids, biomarkers, psychosis

## Abstract

Systemic lupus erythematosus (SLE) is an idiopathic chronic autoimmune disease that can affect any organ in the body, including the neurological system. Multiple factors, such as environmental (infections), genetic (many HLA alleles including DR2 and DR3, and genes including C4), and immunological influences on self-antigens, such as nuclear antigens, lead to the formation of multiple autoantibodies that cause deleterious damage to bodily tissues and organs. The production of autoantibodies, such as anti-dsDNA, anti-SS(A), anti-SS(B), anti-Smith, and anti-neuronal DNA are characteristic features of this disease. This autoimmune disease results from a failure of the mechanisms responsible for maintaining self-tolerance in T cells, B cells, or both. Immune complexes, circulating antibodies, cytokines, and autoreactive T lymphocytes are responsible for tissue injury in this autoimmune disease. The diagnosis of SLE is a rheumatological challenge despite the availability of clinical criteria. NPSLE was previously referred to as lupus cerebritis or lupus sclerosis. However, these terms are no longer recommended because there is no definitive pathological cause for the neuropsychiatric manifestations of SLE. Currently, the treatment options are primarily based on symptomatic presentations. These include the use of antipsychotics, antidepressants, and anxiolytic medications for the treatment of psychiatric and mood disorders. Antiepileptic drugs to treat seizures, and immunosuppressants (e.g., corticosteroids, azathioprine, and mycophenolate mofetil), are directed against inflammatory responses along with non-pharmacological interventions.

## 1. Introduction

Systemic lupus erythematosus (SLE) is a chronic idiopathic autoimmune disease affecting various organs including the central nervous system, presenting as seizures and psychosis. Patients with SLE also present numerous neuropsychiatric manifestations. These neuropsychiatric manifestations are referred to as neuropsychiatric systemic lupus erythematosus (NPSLE). NPSLE affects both the central nervous system (CNS) and the peripheral nervous system (PNS) and can present as various symptoms, such as cognitive dysfunction, organic brain syndromes, delirium, seizures, headache, and psychosis [1].

Some people with NPSLE have problems with their brain and nerves. They may have strokes, confusion, mood disorders, and trouble with thinking and memory. This is called NPSLE, and it affects from 3 to 4 out of 10 people with SLE. Sometimes, NPSLE is the first sign of SLE, and it can happen even when SLE is not very active. Scientists are trying to figure out what causes NPSLE. They think it may have to do with blood clots, antibodies that attack the brain, and proteins that cause inflammation. They also want to know how some chemicals in the body, like TNF, IL-1, IL-6, and IFN-γ, affect the brain and nerves. Some new studies suggest that another chemical, called TWEAK, may play a role in NPSLE in humans and mice [2].

In SLE there are eight types of pleuropulmonary involvement: lupus pleuritis, acute lupus pneumonitis, pleural effusion, shrinking lung syndrome, diffuse alveolar hemorrhage, pulmonary arterial hypertension, interstitial lung disease, and pulmonary embolism [3].

Likewise, the skin is mostly affected by “butterfly rash”, photosensitivity, and vasculitis [4]. SLE affects the heart, including pericarditis and endocarditis. Glomerulonephritis is a renal involvement of SLE [5], as well as diseases of the joints such as arthritis [6,7]. Lymphadenopathy, autoimmune haemolytic anaemia, autoimmune leukopenia, and autoimmune thrombocytopenia [8] are also manifestations of SLE.

When cells fail to die properly, they can trigger autoimmune disease. A faulty ‘death receptor’ called FAS prevents the removal of self-reactive lymphocytes in the body, causing them to accumulate and cause inflammation and organ damage in mice (Fas(lpr/lpr) (mice with a genetic mutation that causes excessive cell growth) and humans. The REL/NF-κB family of proteins controls many aspects of immunity and is also involved in different aspects of autoimmunity [9]. Recurrent infections such as pneumonias [10,11], oral ulcers [12], and discoid lupus are present in numerous individuals with SLE. Gastrointestinal manifestations include lupus mesenteric vasculitis, intestinal pseudo-obstruction, and protein-losing enteropathy [13].

Notably, the presence of neuropsychiatric symptoms (NPS) in SLE patients does not explicitly indicate the cause of SLE. This is because NPS can be comorbid, coincidental, or a complication of SLE treatment, most notably psychotropic drugs such as corticosteroids. NPSLE is further classified as either primary or secondary [1]. Primary NPSLE syndromes result from direct CNS autoimmune inflammatory processes, whereas secondary NPSLE syndromes are caused by indirect complications of SLE such as treatment side effects, CNS infection from chronic immunosuppression, or SLE-related organ damage [14].

Psychosis is an uncommon neuropsychiatric manifestation of NPSLE but can be secondary to long-term, high-dose glucocorticoids. Glucocorticoids are among the mainstay drugs for the treatment of NPSLE, which makes management even more difficult as they are known to have psychiatric side effects and can cause a spectrum of psychiatric symptoms, including mania, psychosis, anxiety, and depression. On initial clinical examination, it may be difficult to differentiate the cause of psychosis as a result of steroids or NPSLE because no single laboratory test is currently available to definitively confirm the diagnosis of NPSLE [15].

There are 19 neuropsychiatric syndromes observed in SLE, as listed by the American College of Rheumatology (ACR) Nomenclature for NPSLE [16]. The ACR’s nomenclature for NPSLE was last revisited in 2021 [12], as shown in Table 1. NPSLE can precede the onset of lupus or occur at any time during its course [17].

Table 1 shows that, although the frequency of NPSLE syndromes varies tremendously, they could be used as qualitative diagnostic criteria if present to confirm the diagnosis of NPSLE. In addition, this should be accompanied by some laboratory findings for SLE, including the presence of antinuclear antibodies (ANA) such as anti-ds DNA antibodies, which are the hallmark of SLE [17,19].

## 2. Most Systemic Lesions Are Due to Loss of Tolerance to Self-Antigens

The exact etiology of SLE remains to be fully delineated; however, most systemic lesions are due to the loss of tolerance to self-antigens, including histone, ribonucleoprotein, double-stranded DNA, antigen Ro (SSA), and antigen La (SSB); direct or indirect damage from autoantibody formation; and the generation of immune complexes (type III hypersensitivity) [20]. This was confirmed by the fact that anti-DNA complexes can be found in many organs such as the kidneys, lungs, and blood vessels. Serum complement levels, which are also measured during the initial workup and disease monitoring, can markedly decrease secondary to consumption and granular deposition. Therefore, low serum complement levels and immunofluorescence which illustrates granular complement deposits in the glomeruli further support the immunological etiology of the disease.

Alterations in B and T cell activation, along with an impaired clearance of apoptotic debris, have also been implicated in SLE histopathology. NPSLE patients showed significantly more microinfarction, macroinfarction, vasculitis, and microthrombi upon histological analysis than SLE patients without neuropsychiatric manifestations [21]. The histopathological analysis of NPSLE patients varies from nonspecific findings of focal vasculopathy to more specific lesions, including C4d- and C5b-9-associated microthrombi and diffuse vasculopathy [22], which often correlate with the clinical syndromes that define NPSLE [23].

## 3. SLE Immunopathogenesis

### 3.1. Apoptosis Cascade and Role of IFN-α in SLE

**Figure 1:**
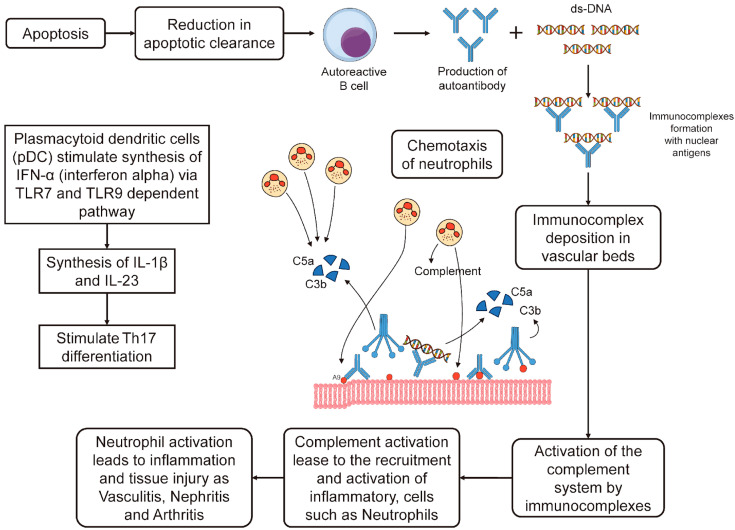
In Systemic lupus erythematosus (SLE), a type III hypersensitivity reaction occurs. This involves the formation of immune complexes that trigger the activation of the complement system. Two components of this system, C3a and C5a, serve as chemotactic factors. They draw neutrophils to the location where the immune complexes are deposited. The activation of these neutrophils results in inflammation and damage to the site, leading to conditions such as vasculitis, nephritis, and arthritis. There may also be other mechanisms at play. Adapted from [24].

A low clearance of apoptotic debris in SLE will lead to an increased formation of autoantigen-antibody complexes by autoreactive B cells. Initially, granulocytes and other innate cells, via pattern-recognition receptors, will recognize apoptotic bodies. The binding of ligands with toll-like receptors (TLRs) activates B-cells, which produce autoantibodies leading to immune complex formation and the activation of the complement system. Plasmacytoid dendritic cells (pDC) stimulate the synthesis of endogenous type I interferon (IFN-α) via a TLR7- and TLR9-dependent pathway. This causes the synthesis of cytokines including (IL)-1β and IL-23 and stimulates Th17 differentiation [24]. Figure 1 shows the type III hypersensitivity mechanism (immunocomplex-mediated) which is involved in the immunopathogenesis of SLE.

### 3.2. Increased Association of HLA System and SLE in a Population

We found, in the Saudi population, that some HLA alleles increased the risk of SLE: HLA-A29 (OR = 2.70; 95% CI = 1.03–7.08; *p* = 0.0035), HLA-B51 (OR = 1.81; 95% CI = 1.17–2.79; *p* = 0.0066), HLA-DRB115 (OR = 1.45; 95% CI = 0.98–2.29; *p* = 0.063), and HLA-DQB106 (OR = 1.67; 95% CI = 1.19–2.36; *p* = 0.0032). On the other hand, HLA-DRB116 reduced the likelihood of the disease (OR = 0.18; 95% CI = 0.02–1.3; *p* = 0.055). HLA-DRB115 haplotypes had a significant link with SLE (OR = 2.01, 95% CI = 1.20–3.68, *p* = 0.008), mainly because of the HLADRB115–DQB106 combination. Our results indicate a relationship between class I and class II MHC (HLA-A29, HLA-B51, HLA-DRB115, and HLA-DQB106) and SLE vulnerability in the Saudi population [25].

### 3.3. Immunopathogenesis of NPSLE

#### 3.3.1. Genetic Factors

**Figure 2:**
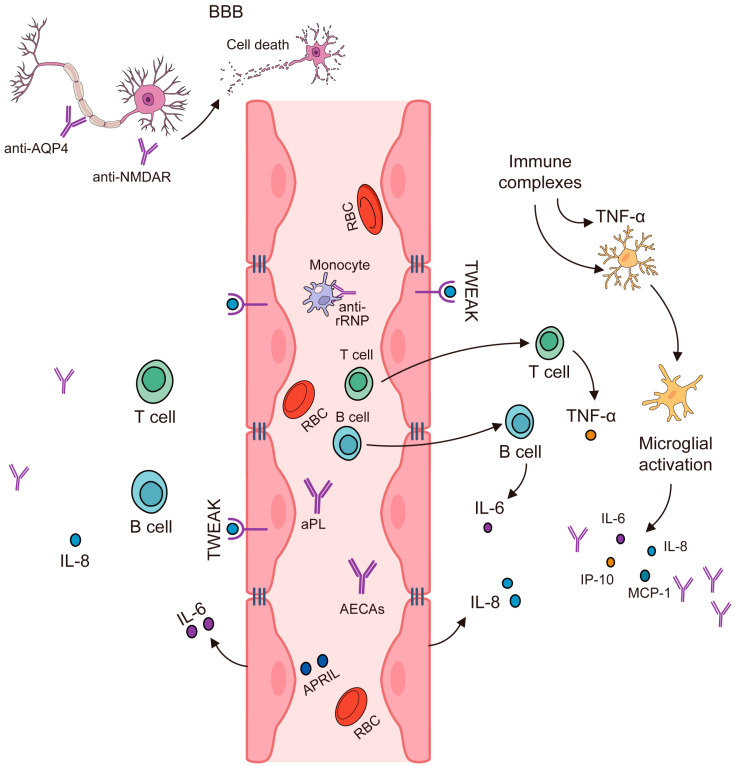
Immunopathogenesis of NPSLE. Modified from [39].

The immunopathogenesis of NPSLE is shown in Figure 2. It is shown to include genetic factors such as the TREX1 gene and HLA-DRB1*04 [12]. The TREX1 gene encodes for TREX1, which is a protein that is expressed extensively and functions as a component of the SET complex in the process of granzyme A-mediated apoptosis, where it degrades single-stranded DNA. The TREX1 gene codes for a 3′-exonuclease 1 protein, which eliminates nucleotides from the 3′ ends of DNA strands, thereby removing unnecessary fragments that might have been produced during DNA replication. The TREX1 gene has been identified to have a role in the regulation of the immune system and in viral infections. Studies have discovered that alterations in this gene are associated with numerous diseases, such as Aicardi-Goutieres syndrome (AGS), systemic lupus erythematosus (SLE), familial chilblain lupus (FCL), retinal vasculopathy, and cerebral leukodystrophy. A common feature in these autoimmune diseases is the frequent detection of antibodies to double-stranded DNA (dsDNA) [26,27,28]. The homodimer TREX1R114H/R114H exhibits impaired dsDNA and ssDNA degradation functions and does not noticeably hinder the TREX1WT enzyme. On the other hand, the heterodimer TREX1WT/R114H retains functional dsDNA degradation activity, which corroborates the recessive genetic nature of TREX1 R114H in AGS and the proposed mechanism of the TREX1 exonuclease [28].

As previously mentioned, among the genetic factors for NPSLE, HLA-DRB1*04 could play a critical role. In Malay SLE patients, HLA-DRB1*04 was found to have a significant correlation with lupus nephritis, characterized by high levels of anti-ds DNA Ab, and arthritis. Further analysis of the association of HLA-DRB1*04 with clinical and biological factors showed that it had a significant correlation with the Systemic Lupus Erythematosus Disease Activity Index (SLEDAI) scores, C-reactive protein (CRP) in the blood, anti-nuclear antibody (ANA), and total protein in the urine. It was also observed that SLE carriers possessing the HLA-DRB1*04 allele had a significant correlation with elevated levels of cytokines such as IL-17F and GM-CSF [29].

In summary, some the genetic factors involved in SLE are:

- Transcriptomic data analysis has revealed several pathways and immune responses that are associated with SLE, such as interferons, T cell differentiation, complement pathways, and coagulation;
- Eight genes (SOCE, CXCL8, MMP9, IL1B, JUN, TNF, NFKBIA, and FOS) are up-regulated in SLE and have interactions with different pathways. These genes are also linked to SNPs that are identified by GWAS;
- Several other genes with known SLE-related variations are detected by integrating GWAS and pathway analysis, such as TYK2, SH2B, C5, IL2RA, IRF5, FCGR2A, TNFAIP3, STAT4, LYN, IL7R, and HLA-DRB;
- One of the relevant pathways that is identified by pathway-based analysis is the TSLP signaling pathway, which is connected to rs7574865, LYN, STAT4, and IL7R;
- The results of this study have increased the number of candidate genes for SLE and have shown potential pathways and methods for gene discovery. Finding the key genes would help to understand the mechanisms of SLE [19,30,31,32].

#### 3.3.2. Comorbidities

Lupus nephritis (LN) and NPSLE are both severe manifestations of SLE, a chronic autoimmune disease [33]. LN is a type of glomerulonephritis mediated by immune complex deposition at glomerular sites, representing one of the severe major organ involvements seen in SLE. On the other hand, NPSLE refers to a series of neurological and psychiatric symptoms directly related to SLE that involve the central and peripheral nervous system [33].

The comorbidity of LN and NPSLE in SLE patients can be explained by the systemic nature of SLE, which can affect any organ, including the kidneys and the nervous system [34]. The immune response in SLE patients can lead to inflammation and damage in various parts of the body, including the kidneys (leading to LN) and the nervous system (leading to NPSLE) [29].

Moreover, neuropsychiatric symptoms, whether causally associated or comorbid, negatively impact the quality of life of patients with SLE. In addition, these symptoms appear to identify patients with a higher mortality than those without neuropsychiatric symptoms. It is important to note that, while the attribution of neurologic symptoms to SLE may influence decisions about disease-modifying treatments, the timely recognition of neuropsychiatric comorbidity in SLE patients is also important to provide appropriate symptomatic management [34]. Therefore, understanding the comorbidity of LN and NPSLE in SLE patients is crucial for their management and treatment.

The clinical manifestations depend on environmental, immunological, hormonal, and genetic factors. The blood–brain barrier (BBB) can breach through multiple mechanisms. These include autoimmune processes, such as immune complex deposition and pathologic cytokine-mediated destruction, and environmental factors, such as smoking and hypertension [35]. There are no specific abnormalities noted on the brain images of patients with NPSLE, and some may even have normal findings, nonspecific white matter changes, or atrophy [14].

#### 3.3.3. Summary of NPSLE Immunopathogenesis

In summary, NPSLE is a complex condition with a multifaceted pathogenesis involving genetic factors, cytokines, immune cells, and environmental factors [36], as shown in Figure 2.

Genetic Factors: Certain genes have been associated with the development of NPSLE. For instance, specific alleles of the human leukocyte antigen (HLA) genes have been linked to an increased risk of developing NPSLE [36,37,38]. We already refer to the TREX1 gene as a genetic factor in the immunopathogenesis of this autoimmune disorder [12].

Cytokines: Cytokines, which are signaling molecules in the immune system, play a crucial role in the pathogenesis of NPSLE. They mediate inflammation and immune responses, contributing to the neurological and psychiatric symptoms observed in NPSLE. For example, increased levels of certain cytokines (like IFN-y, IL-17F, IL-21, IL-18, GM-CSF, and VEGF) have been observed in SLE patients [36].

Immune Cells: The role of immune cells in NPSLE is significant. Autoantibodies produced by B cells can cross the blood–brain barrier (BBB) and bind to brain tissue, causing inflammation and damage [40]. T cells also contribute to the pathogenesis of NPSLE by producing pro-inflammatory cytokines [39], as shown in Figure 2.

Environmental Factors: Environmental factors such as infections, stress, smoking, and exposure to ultraviolet light can trigger the onset of NPSLE in genetically predisposed individuals [36].

Pathogenesis: The pathogenesis of NPSLE is thought to involve a combination of the above factors. The presence of certain autoantibodies (like anti-ribosomal P, anti-NR2, and anti-16/6 Id antibodies) and brain cytoplasmic ribonucleic acid (BC RNA) antibodies, which disrupt normal brain function, have been found in NPSLE patients [40,41]. These antibodies, along with the cytokines, can cause neurocognitive symptoms [18,36,41,42]. Additionally, the dysfunction of the BBB and vascular lesions may contribute to the development of NPSLE [36]. It is important to note that the exact mechanisms are still being researched, and the pathogenesis likely involves a complex interplay of these factors [36,39]. Other factors to take into consideration regarding the immunopathogenesis of NPSLE are the presence and interaction of chemokines with the innate and acquired immune system, the existence of lymphocytic infiltrate, the activation of endothelial cells in the brain, the activation of microglial cells, and neuronal cell death by apoptosis [42].

### 3.4. IL-2, IL-10, and IFN-γ Produced by T-Helper Cells Are Elevated in NPSLE

IL-2, IL-10, and IFN-γ produced by T-helper cells are elevated in NPSLE, and this is directly related to autoimmune and pro-inflammatory states [18,43]. The neuropsychiatric manifestations of SLE are likely due to antibodies that react with neurons either directly or indirectly via the activation of other neural cells and cross the BBB due to its disruption. Other immunological factors, such as cytokine-mediated CNS toxicity, may also play a role. Some of the cytokines found to be elevated in patients with NPSLE include IL-2, IL-10, and IFN-γ produced by T-helper cells [44]. The pathogenesis of compromised BBB integrity is not yet fully understood. However, once they enter the CNS, antibodies and cytokines can cause clinical effects. Therefore, clinically useful biomarkers must be identified [37].

### 3.5. Noninflammatory or Thrombotic/Ischemic Vascular Injury

Noninflammatory complications are associated with vascular thrombosis and hemorrhage. The thrombosis of large and small intracranial vessels can occur due to immune complex damage, antibody-mediated damage, complete deposition, accelerated atherosclerosis, or leucoagglutination [1,18,45]. Cerebral vasculitis has also been associated with NPSLE. CNS vasculopathy via antibodies, such as anti-phospholipid antibodies, may damage the BBB and allow CNS immune complex deposition, resulting in NPSLE. However, CNS inflammatory vasculopathy is rare, and non-inflammatory vasculopathy is more commonly observed [46].

While this classification is useful for descriptive purposes, both inflammatory and non-inflammatory complications can occur [47]. These manifestations of NPSLE are classified based on the 19 syndromes described by the ACR; however, they are not yet fully understood. The predominant inflammatory syndromes can result from the generation of pathological autoantibodies associated with cytokine-mediated damage [44].

## 4. Autoantibodies Can Lead to Neuronal Damage in NPSLE

An overriding feature of SLE is the involvement of the immune system and the production of autoantibodies. The immune mediators of NPSLE are quite extensive and include autoantibodies, cytokines, and chemokines. Autoantibodies can lead to neuronal damage and promote the pathogenesis of NPSLE. More than 116 antibodies have been reported for SLE, and at least 11 brain-specific and 9 systemic antibodies have been associated with NPSLE [36,48]. However, none of these autoantibodies have definitive implications in the pathogenesis of NPSLE, and their association remains controversial [49].

Brain-specific antibodies associated with NPSLE include anti-neuronal Abs, brain-reactive Abs (BRAA), Anti-N-methyl-D-aspartate receptor Abs (NMDA), anti-microtubule-associated protein 2 Abs (MAP-2), anti-neurofilament Abs (ANFA), anti-ganglioside Abs (AGA), anti-central nervous system tissue (CNS) Abs, anti-brain-synaptosomal Abs, anti-triosephosphate isomerase (TPI) Abs, anti-glial fibrillary acidic protein (GFAP) Abs, and anti-serum-lymphocytoxic Abs (LCA) [50]. Systemic antibodies include antiphospholipid (aPL)/cardiolipin (aCL) Abs, lupus anticoagulant (LAC), anti-beta 2- glycoprotein I (2GPI) Abs, anti-ribosomal P Abs (anti-P), anti-Ro Abs, anti-Sm Abs, antiendothelial Abs (AECA), anti-serine proteinase (anti-PR3/C-ANCA) Abs, and anti-Nedd5 Abs [50].

### 4.1. Antiphospholipid Antibodies (β2-Glycoprotein 1, Cardiolipin Anticardiolipin (Anti-CL) and Lupus Anticoagulant (LA)

The aPL antibodies have an affinity to, and therefore target, anionic phospholipids, including β2GPI (rather than being against anionic phospholipids, which their name would suggest [51]) in the plasma membrane that regulates the blood clotting cascade [52]. The subsequent activation of procoagulants promotes thrombosis and cerebral infarction [53], and aPL antibodies have been identified for focal and diffuse NPSLE symptoms such as cognitive dysfunction [54], seizures [55], stroke, transient ischemic attack [56,57], movement disorders, chorea [58,59], and myelopathy [60].

### 4.2. Ribosomal P Protein (Anti-Ribosmal P Ab)

Anti-ribosomal P (anti-Rib-P) antibodies are specific serological markers observed in patients [61]. Anti-ribosomal-P antibodies are located at the carboxy-terminal end of the 60S subunit of ribosomes and target three phosphorylated proteins, P0, P1, and P2 [62]. Anti-ribosomal-P-antibodies are believed to breach the BBB, penetrate neuronal cells, and inhibit protein synthesis [63,64,65]. Antibodies against ribosomal-P proteins are associated with diffuse NPSLE, psychosis, and clinically significant depression in patients [66,67,68]. Their presence may be a risk factor for the development of NPSLE [69] and a predictor of psychosis in patients already diagnosed with NPSLE [70]. These antibodies may also be associated with complications of the peripheral nervous system [71]. In animal studies, depressive behavior was noted when anti-ribosomal P antibodies were introduced into the cerebral ventricles [72]. These antibodies cross-react with the neuronal surface P antigen on the membranes of neurons in the hippocampus and can manifest as clinical depression [73,74]. The anti-Rib-P antibody can also cross-react with NMDA receptors, resulting in psychosis [75], although the presence of anti-rib-P is not always associated with NPSLE manifestations [76]. Therefore, the clinical significance of anti-rib-P antibodies remains controversial.

### 4.3. Anti-Human N-Methyl-D-Aspartate Receptor Abs (Anti-NMDA)

The NMDA receptor is an ionotropic glutamate receptor in the CNS that is responsible for synaptic plasticity and memory [77]. The NR2A and NR2B subunits are found in the hippocampus, amygdala, and hypothalamus [78]. NMDA receptors are tetramers composed of NR1 subunits and two of the four NR2 (A–D) subunits [79]. Anti-NMDAR encephalitis is an autoimmune neurological condition associated with SLE; however, its pathophysiology is not fully understood [80,81]. Anti-NR2 antibodies cross-react with anti-double-stranded DNA antibodies [82]. Anti-NR2 antibodies can enter the CNS via intrathecal IgG synthesis or by breaching the BBB [83]. The severity of BBB damage plays a significant role in diffuse NPSLE syndromes, including the potential acute confusional state, because it allows large titers of anti-NR2 to enter the CNS [84].

In NPSLE patients, anti-NR2 antibodies pathologically bind to the extracellular domains of the NR2A and/or NR2B subunits of the NMDA receptor. These autoantibodies have a much higher sensitivity to the NR2A subunit, resulting in excessive activation of the NMDA receptor [85]. Pathological NMDA receptor activation in patients has been found to manifest as epilepsy, encephalitis, schizophrenia, mania, stroke, and cognitive impairment [78,86].

### 4.4. Microtubule-Associated Protein (Anti-MAP-2 Ab)

MAP-2 is a cytoskeletal protein expressed primarily in neuronal cells that is responsible for microtubule nucleation and stabilization, and regulates organelle transport protein kinases involved in signal transduction [87,88]. Anti-MAP-2 antibodies are associated with neuronal injury and death and are significantly elevated in the CSF of patients with NPSLE [89]. Anti-MAP-2 antibodies are associated with neuropsychiatric symptoms, such as psychosis, schizophrenia [90], bipolar disorder [91], major depression [92], seizures, neuropathy, and cerebritis [42].

### 4.5. U1 Ribonucleoprotein (Anti-U1RNP Ab)

Anti-UIRNP antibodies are observed in autoimmune conditions such as mixed connective tissue disease (MCTD), systemic sclerosis (SSc), and systemic lupus erythematosus (SLE) [93]. The small nuclear ribonucleoproteins (snRNP) are RNA–protein complexes found in abundance in the nucleus and are involved in the processing of pre-mRNA and other proteins comprising the spliceosome [94]. Anti-U1RNP antibodies react with one or more of the three proteins (70-kD, A, and C) that are specifically present in the U1 RNP complex to form U1 small nuclear ribonucleoprotein (snRNP) [95]. The snRNP is a target of autoreactive B and T cells in several rheumatic diseases, including SLE [96]. Anti-U1 RNP antibodies range from 3 to 69 percent in patients with SLE [97]. Anti-U1RNP Ab is associated with NPSLE manifestations such as anxiety, seizures, and CVD [98].

### 4.6. Structural Endothelial Proteins (AECA)

Endothelial cells (ECs) are found on the inner walls of blood vessels and form a layer of cells referred to as the endothelium. Endothelial cells have not been previously considered as components of the immune system. ECs are important for regulating blood pressure and play important roles in coagulation, fibrinolysis, angiogenesis, and immune cell activation via both physiological and pathological processes [99]. The modulation of endothelial cells via the adaptive and innate immune systems plays an integral role in autoimmune diseases, as endothelial cells promote chronic inflammation via angiogenesis, attracting immune cells, and antigen presentation [100]. Anti-endothelial cell antibodies (AECA) are a heterogeneous group of autoantibodies directed against structural endothelial proteins, along with antigens on endothelial cells [101]. The activation of ECs leads to increased leukocyte adhesion, the activation of coagulation, and vascular thrombosis in a dose-dependent manner [102]. The pathologic activation of ECs results in endothelial injury and an increased risk of complications, such as atherosclerosis and vascular thrombosis, which are the most common causes of premature mortality in patients with SLE [103].

### 4.7. Triosephophate Isomerase (Anti-TPI Ab)

Triosephosphate isomerase (TPI) is a glycolytic enzyme found in neuronal and red blood cells that are involved in the interconversion of dihydroxyacetone phosphate (DHAP) and glyceraldehyde-3-phosphate (G3P) [104]. Anti-TPI antibodies have been associated with NPSLE with a higher frequency of aseptic meningitis and elevated serum IgG levels in anti-TPI-positive NPSLE patients compared to anti-TPI negative NPSLE patients [105]. Anti-TPI antibodies likely breach the BBB via meningeal inflammation. Anti-TPI antibodies form immune complexes in the CSF and activate the classical complement system, contributing to the pathogenesis of NPSLE [106].

### 4.8. Glyceraldehyde-3-Phosphate Dehydrogenase Antibodies (Anti-GAPDH)

GAPDH is a glycolytic enzyme that also plays a role in cell membrane fusion, microtubule bundling, nuclear RNA export, and DNA replication and repair [107]. Anti-GAPDH antibodies have been associated with increased disease activity, increased inflammation, and increased intracranial pressure in NPSLE patients [108]. GAPDH antibodies have been found in at least 50% of NPSLE patients with schizophrenia and major depression and may be a future potential biomarker of NPSLE [109]. A significant positive relationship between the levels of anti-GAPDH antibodies in the serum and detrimental cognitive and mood conditions (such as schizophrenia and major depression) in patients with SLE has been reported. The levels of anti-GAPDH antibodies were found to be higher in SLE patients exhibiting psychotic symptoms compared to those without such symptoms [109]. This finding is further supported by a study conducted by Sun and collaborators, which discovered that the levels of anti-GAPDH antibodies in the serum were significantly increased in NPSLE patients and were correlated with increased SLEDAI-2K, ESR, IgG, and IgM [108].

Anti-GAPDH antibodies are used in the immunodetection of the protein encoded by the GAPDH gene. GAPDH is an enzyme that catalyzes the sixth step of glycolysis and serves to break down glucose for energy and carbon molecules. In addition to its role in metabolism, GAPDH is involved in the initial stages of apoptosis and the oxidative stress response where GAPDH is translocated to the nucleus. Such actions may reflect the role of GAPDH in DNA repair or as one nuclear carrier for apoptotic molecules. GAPDH has also been found to bind specifically to proteins implicated in the pathogenesis of a variety of neurodegenerative disorders [110,111].

### 4.9. Anti-Aquaporin Four Antibodies (NMO-IgG/AQP4-Ab)

Anti-AQP4 antibodies, also known as NMO-IgG, have been identified in patients with NPSLE [112]. These antibodies target the Aquaporin 4 (AQP4) water channel protein, which is predominantly found in the central nervous system. In one study, it was found that these antibodies were present in a patient with transverse myelitis, a rare but serious complication of SLE [1,112]. However, they were not detectable in NPSLE patients with other neurological manifestations. This suggests that testing for NMO-IgG/AQP4-Ab positivity should be considered in patients presenting with SLE and TM [1,112].

### 4.10. Anti-Endothelial Cell Antibodies (AECAb)

Anti-endothelial cell antibodies (AECAb) are autoantibodies that target endothelial cells, which are the cells that line the interior surfaces of blood vessels [36]. In the context of neuropsychiatric systemic lupus erythematosus (NPSLE), these antibodies have been associated with various pathogenic mechanisms [2,113]. AECAb has been implicated in inducing a proadhesive and proinflammatory endothelial phenotype through nuclear factor kappa B (NF-κB) activation, with the involvement of an autocrine loop of interleukin-1β secretion [36].

AECAb may contribute to the dysfunction of BBB, a layer of cells that prevents harmful substances in the blood from crossing into the brain. The presence of AECAb in NPSLE patients could potentially lead to cerebrovascular ischemia as a result of a generally prothrombotic state [36].

### 4.11. Anti-Ubiquitin Carboxyl Hydrolase L 1 Antibodies (Anti-UCH-L1 Ab)

Anti-UCH-L1 Abs have been studied as potential biomarkers for NPSLE. In particular, the autoantibody against the amino acids 58–69 of UCH-L1 (UCH58-69) has shown significant diagnostic power in distinguishing NPSLE patients from SLE patients without neuropsychiatric symptoms. The specificity and sensitivity of anti-UCH58-69 were found to be 92.3% and 37.5%, respectively. Increased serum levels of anti-UCH58-69 were associated with an increased disease severity, suggesting that this autoantibody could be a novel serum biomarker for the non-invasive diagnosis of NPSLE. This might be applicable for early screening and diagnosis of NPSLE. However, it is important to note that these findings are based on research studies, and further validation is needed before these antibodies can be used in clinical practice [39,114]. In a study conducted by Li et al. in 2019, it was found through a randomized controlled trial that antibodies against UCH-L1 could serve as a reliable biomarker in the cerebrospinal fluid (CSF) for diagnosing NPSLE. The study also found that the levels of UCH-L1 in the CSF could indicate the severity of NPSLE [115]. In a similar vein, a recent study on autoantibodies showed that the autoantibody targeting the amino acids 58–69 of UCH-L1 (UCH58-69) demonstrated a high level of specificity and diagnostic significance in differentiating NPSLE patients from SLE patients without neuropsychiatric symptoms. The study further revealed that the levels of anti-UCH58-69 in the serum were considerably higher in NPSLE patients compared to SLE patients without neuropsychiatric symptoms, and these levels were found to be associated with the severity of the disease [36]. Table 2 illustrates autoantibodies associated with NPSLE.

Autoantibodies against autoantigens were detected in the sera of NPSLE patients, as well as in the CSF. However, the authors did not compare the prevalence of these autoantibodies in the sera of NPSLE and non-NPSLE patients, so it is not clear if they are specific to NPSLE. Based on their results, the autoantibody that has the highest possibility of being a marker of NPSLE is anti-SS-A, because it showed a significantly higher positive rate in the CSF of NPSLE patients than in the CSF of non-NPSLE patients, and it was also related to neuropsychiatric syndromes of the central nervous system in SLE patients. The authors used a human proteome microarray to screen for autoantibodies in the cerebrospinal fluid (CSF) of patients with neuropsychiatric systemic lupus erythematosus (NPSLE), a subtype of SLE that affects the nervous system. They identified autoantigens that were specifically associated with NPSLE, and found that they were enriched for functions involved in neurological diseases. They also found 22 autoantigens that were shared by NPSLE and non-NPSLE patients, and found that they were enriched for functions involved in inflammatory responses. They validated some of the candidate autoantigens using a focused autoantigen microarray and western blot, and confirmed that anti-SS-A and anti-PCNA autoantibodies were significantly associated with NPSLE. They also found that the titers of anti-RPLP2 and anti-SS-A autoantibodies in CSF and serum specimens were significantly correlated, suggesting that they leaked from the blood due to the compromised BBB [116].

## 5. Investigations

NPSLE diagnosis is achieved on a case-by-case basis based on the constellation of clinical signs and symptoms, along with laboratory, electrophysiological, neuroimaging, and histopathological findings, which can also aid in the diagnosis [117,118].

### 5.1. Biomarkers

Anti-nuclear antibodies (ANAs) are positive for most patients with SLE. However, ANAs are also found in the healthy general population and have a low specificity. Therefore, ANA titers cannot be used to diagnose SLE, and must be followed up with specific antibodies. However, ANAs have high sensitivity; therefore, the lack of this antibody in laboratory work makes SLE unlikely, and other diagnoses should be considered. To further complicate the diagnosis, cases of ANA-negative SLE have been reported; therefore, clinical suspicion is of the utmost importance, and once ANAs are shown to be positive, follow-up tests for specific antibodies should be performed. These antibodies include anti-dsDNA, anti-Smith, anti-Ro/SSA, anti-La/SSB, and U1 ribonucleoprotein (RNP) antibodies. Anti-dsDNA has a very high specificity (of >95%) for SLE, and its presence confirms the diagnosis. Anti-Smith antibodies have an even higher specificity (of >90%), and their presence confirms the diagnosis. However, anti-dsDNA and anti-Smith antibodies are observed in only 70% and 30% of patients with SLE, respectively. Therefore, negative anti-dsDNA and anti-Smith antibodies should not rule out the diagnosis of SLE if there is a high clinical suspicion [119,120,121]. Anti-Ro/SSA and anti-La/SSB antibodies were observed in only 30% and 20% of patients with SLE [122], respectively. These antibodies are observed in >90% of patients with Sjögren’s syndrome. Their presence in patients with SLE warrants workup for secondary Sjögren’s syndrome. Pregnant mothers should also be advised about the possibility of a congenital heart block. Anti-U1 RNP antibodies are present in approximately 25% of patients with SLE. Their presence is typically observed in mixed connective tissue diseases. They are almost always found concurrently with the anti-Smith antibodies [122,123].

A complete blood count with a differential diagnosis can also be performed, as it may reveal thrombocytopenia, anemia, and/or leukopenia. Inflammatory markers, such as ESR and CRP, may also be elevated. Creatinine levels can be raised in patients with renal involvement along with an elevated urine protein-to-creatinine ratio. Urine analysis can reveal proteinuria, hematuria, and cellular casts. Changes C3, C4, and CH50 levels can also be found, as a decrease in their levels suggests complement activation and consumption, and can be related to disease activity [124]. Antiphospholipid antibodies (e.g., lupus anticoagulant, anticardiolipin antibodies, and anti-beta2-glycoprotein 1, are associated with a higher likelihood of thrombotic events. Anti-ribosomal P protein antibodies have a high specificity but a low sensitivity for SLE [125,126]. They are present in a minority of SLE patients; however, some studies have suggested that this antibody is a marker of CNS disease, although this is controversial. Testing for anti-ribosomal P protein antibodies has limited clinical value; however, there is an association between their presence and neuropsychiatric manifestations of SLE, especially psychosis.

### 5.2. Serum and CSF Analyses

The 2019 EULAR/ACR classification criteria for SLE and the SLE Disease Activity Index (SLEDAI) include several serological parameters along with signs, symptoms, radiological features, and histologic and pathological findings for the classification of SLE [127,128]. However, in the absence of concurrent systemic inflammation, these parameters often cannot be used to predict neuropsychiatric disease activity [129].

CSF analysis can be useful in excluding other etiologies; however, the findings are often nonspecific. CSF analysis can reveal nonspecific findings of inflammation such as elevated total protein, elevated IgG, pleocytosis, and mildly reduced CSF glucose levels. Pleocytosis observed in CSF analysis is associated with ‘lupus psychosis’ and delirium [130]. Pleocytosis has been reported in approximately 20% of NPSLE cases and is typically at a low level, although it has been reported with white cell counts greater than 100 cells/μL [131,132]. Protein elevation may be observed in 20–30% of NPSLE patients, with levels ranging from 1 g/L to greater than 2 g/L [133,134]. CSF abnormalities are seen in 30–40% of cases and, therefore, do not provide a reliable differentiation of NPSLE from non-neuropsychiatric SLE patients [135].

### 5.3. Biomarkers in NPSLE

Ni et al., in 2023, discussed the discovery of novel biomarkers for improving the diagnostic efficiency for NPSLE. The study used a quantitative planar protein antibody microarray to screen 1000 proteins in cerebrospinal fluid from controls, systemic lupus erythematosus (SLE, non-NPSLE) patients, and NPSLE patients. Differentially expressed proteins (DEPs), as candidate biomarkers, were developed into a custom multiplexed protein antibody array for further validation in a larger independent cohort. Subsequently, they used least absolute shrinkage and selection operator regression (LASSO) analysis and multivariable logistic regression analysis for optimizing their feature selection and constructing a diagnostic model. A receiver operating characteristic curve (ROC) was generated to assess the effectiveness of the models. The study identified five DEPs as biomarkers for NPSLE, including TCN2, KLK5, CST6, Trappin-2, and L-selectin. The diagnostic model included three hub proteins (CST6, KLK5, TCN2) and was the best at discriminating NPSLE from SLE patients. These CSF biomarkers were also highly associated with the disease activity. In addition, there were six molecules with remarkable changes in NPSLE CSF and the hippocampus, which indicated the consistency of the environment in the brain and their promise as molecular targets in the pathogenesis of NPSLE [136].

## 6. NPSLE Complications Caused Directly by NPSLE or the Treatment

Drug-induced psychosis can occur at any time during the treatment of NPSLE. The symptoms include anxiety, agitation, irritability, and insomnia. More severe symptoms, such as mania, psychosis, and depression, can also occur. Steroid-induced psychosis is thought to be dose-dependent and more likely to occur in patients in long-term therapy [137].

### 6.1. Steroid Induced Psychosis

In patients receiving long-term steroid therapy for the management of systemic lupus erythematosus, the diagnosis becomes more complicated as the differentiation of a neuropsychiatric flare from steroid-induced psychosis is often clinically difficult at the initial presentation [138,139]. Steroid-induced psychosis occurs due to abnormalities in the hypothalamic–pituitary–adrenal axis [140]. Exogenously administered steroids lead to the suppression of steroid secretion via the adrenal glands and eventual atrophy, resulting in disturbances in cortisol levels.

The imbalance in glucocorticoid receptor stimulation can lead to cognitive impairment and psychiatric disturbances such as psychosis [140,141].

### 6.2. Progressive Multifocal Leukoencephalopathy (PML)

Progressive multifocal leukoencephalopathy (PML) is caused by reactivation of the JC virus in immunosuppressed individuals with SLE and/or drug therapy with cyclosporine and methylprednisolone [142,143].

## 7. Management of NPSLE

The validity of biomarkers for systemic lupus erythematosus that are used for making clinical decisions is limited. The lack of reliable and specific biomarkers for NPSLE negatively affects the current and future e management of patients with SLE [144]. Specific treatment modalities depend on whether the symptoms are due to inflammatory, non-inflammatory, or thrombotic reasons [145].

**Figure 3:**
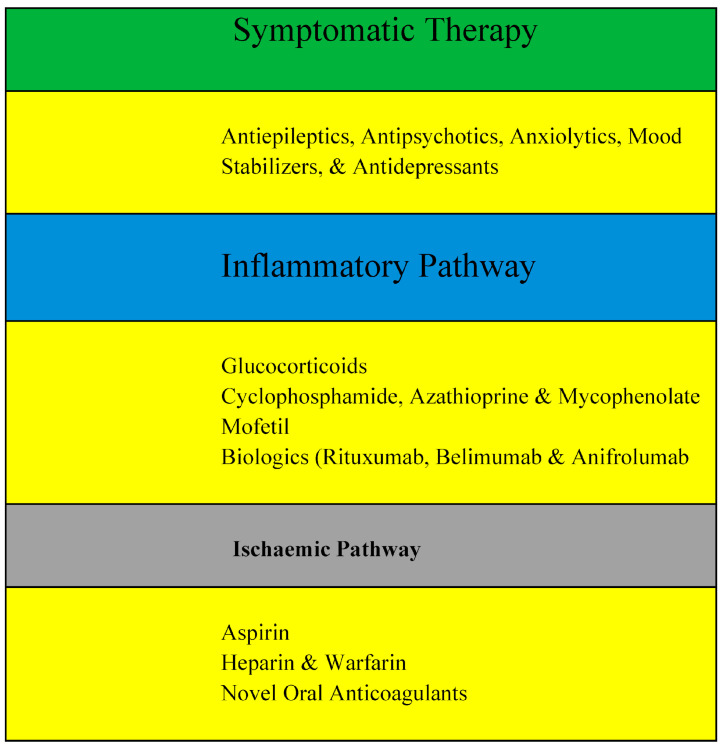
Management for patients with NPSLE. Modified from [39].

The management of NPSLE can be challenging due to the complexity of its pathogenesis, difficulty in its accurate diagnosis, and a lack of clinical trials in NPSLE. The current treatment options for NPSLE are usually derived from observational studies and refer to the experience of the treatment of other SLE subtypes, such as lupus nephritis and similar neuropsychiatric disorders, as shown in Figure 3. The management of NPSLE has two main goals. The first goal is to provide symptomatic therapy, which includes administering antiepileptics for seizures, and anxiolytics, antidepressants, mood-stabilizers, or antipsychotics as appropriate. Neurotrophic and neuroleptic agents are generally adopted in cases with peripheral nervous system involvement. The second goal is to treat the underlying SLE process based on whether the pathogenesis is primarily related to an inflammatory or ischemic disease pathway [18,39].

### 7.1. Symptomatic Therapy

#### 7.1.1. Antiepileptics

Antiepileptic treatment is needed for NPSLE patients with seizures, especially those with high-risk features, such as a second seizure, evidence of brain injury, and focal neurological deficits. Generalized and recurrent seizures warrant the use of antiepileptic drugs such as phenytoin and barbiturates. Partial complex seizures can be managed using drugs such as carbamazepine, valproic acid, and gabapentin. Patients with generalized convulsive status epilepticus (GCSE) require immediate treatment to prevent neurological injury and death. After treatment and stabilization, neuroimaging should be performed as in non-NPSLE patients to evaluate any underlying structural abnormality, hemorrhage, or area of ischemia. Patients with seizures who do not return to normal consciousness can undergo continuous electroencephalography to rule out non-convulsive status epilepticus, and there is no consistent evidence-based therapy for cognitive dysfunction in SLE patients. This is likely because cognitive dysfunction has been associated with many psychosocial factors such as fatigue, sleep deprivation, depression, and anxiety. However, antidepressants and psychotherapy may improve symptoms in patients with comorbid depression. Antimalarial drugs are commonly used in patients with SLE, and there is evidence supporting their use in patients with NPSLE. Antimalarials have been demonstrated to reduce the risk of CVD and antiphospholipid antibody titers. Another benefit of antimalarials is their antithrombotic effect. Antimalarials have also been shown to protect against seizures. However, antimalarial drugs are rarely known to cause psychosis, and chloroquine is associated with epilepsy in patients with a history of epilepsy. The LUMINA study showed a protective role of hydroxychloroquine and time against NPSLE manifestation, but it may have been confounded by indication since patients with a milder disease were more likely to be treated with this drug [39,138,145,146].

#### 7.1.2. Antipsychotics in NPSLE

Antipsychotics, in the treatment of NPSLE, are used to treat psychosis, agitation, and catatonia, which are common and severe manifestations of NPSLE. Antipsychotics can also help reduce inflammation and modulate immune responses in the brain. The choice of antipsychotic depends on the type and severity of the NPSLE symptoms, the underlying pathophysiology, and the patient’s response and tolerance. Second-generation antipsychotics (SGAs) are preferred over first-generation antipsychotics (FGAs) because they have fewer extrapyramidal side effects and a lower risk of neuroleptic malignant syndrome. Clozapine, an SGA, may be the safest option for NPSLE, followed by quetiapine. Aripiprazole, another SGA, has partial dopamine agonist effects and may be useful for NPSLE patients with Parkinsonism or dopamine deficiency. Antipsychotic monitoring is essential to assess the efficacy and safety of the treatment, as well as to prevent or manage adverse effects, such as metabolic syndrome, QTc prolongation, agranulocytosis, and infection. The antipsychotic dose should be adjusted according to the clinical response and the pharmacokinetic properties of the drug. Antipsychotic discontinuation should be gradual and cautious, especially if the patient has a history of NPSLE recurrence [22,145,147].

#### 7.1.3. Anxiolytics in NPSLE

Anxiolytics are a class of medication that is used to alleviate anxiety, and they can play a role in managing NPSLE. This pathology is a complex condition that can result in a broad range of psychiatric syndromes such as psychosis, mood disorders, acute confusion, and cognitive dysfunction. Anxiolytics work by modulating the activity of neurotransmitters in the brain, and they can be particularly useful in managing anxiety disorders that can occur in patients with NPSLE. The choice of anxiolytic medication and the dosage will depend on the specific symptoms, the severity of the disease, and the patient’s response to medication [22,145,147,148,149].

#### 7.1.4. Mood Stabilizers in NPSLE

Mood stabilizers are a class of medication that can help regulate mood swings and reduce symptoms of mania and depression. They are often used to treat bipolar disorder, but they may also be helpful for some people with neuropsychiatric systemic lupus erythematosus (NPSLE). Mood stabilizers are part of the European League Against Rheumatism (EULAR) recommendations for the management of NPSLE, especially for mood disorders, psychosis, and seizures. A 2015 comparative study compared the EULAR recommendations with the usual care in two European centers and found good concordance between them for the diagnosis and treatment of NPSLE. The study also identified some issues that need further investigation, such as the overutilization of brain MRI, the suboptimal evaluation of cognitive dysfunction, and the frequent use of immunosuppressives in cerebrovascular disease [149]. Some of the mood stabilizers that may be used for NPSLE include:

Lithium: This is the oldest and most established mood stabilizer for bipolar disorder. It can help prevent mood episodes and reduce the risk of suicide. However, it can also cause side effects such as weight gain, tremors, thyroid problems, and kidney damage. Regular blood tests are needed to monitor the lithium level and avoid toxicity [150].

Antiepileptics: These are medications that were originally used to treat seizures, but they can also stabilize a patient’s mood and prevent manic or depressive episodes. Some examples are valproate, lamotrigine, carbamazepine, and topiramate. They can have different side effects depending on the drug, such as weight changes, rash, liver problems, blood count abnormalities, and birth defects [151].

Antipsychotics: These are medications that are mainly used to treat psychosis, but they can also have mood-stabilizing effects and help with agitation, insomnia, and anxiety. Some examples are olanzapine, quetiapine, risperidone, aripiprazole, and lurasidone. They can cause side effects such as weight gain, diabetes, high cholesterol, movement disorders, and sedation [152].

The choice of mood stabilizer for NPSLE depends on several factors, such as the type and severity of the symptoms, the presence of other medical conditions, the potential for drug interactions, the patient’s preference, and the doctor’s experience. Mood stabilizers should be used with caution and under close supervision by a rheumatologist and a psychiatrist [18,36,39,149].

### 7.2. Inflammatory Pathway

#### 7.2.1. Glucocorticoids

NPSLE is a complex disease that can be challenging to manage. The use of glucocorticoids and other immunosuppressants has been studied as a potential treatment option for NPSLE. A study by Monahan et al., in 2023, investigated the short-term and long-term outcomes of inflammatory NPSLE with immunosuppressive treatment. The study found that the outcome of inflammatory NPSLE after immunosuppressive treatment is generally good, with improvement in neuropsychiatric symptoms occurring in approximately 70% of events. The most common neuropsychiatric manifestation was cognitive dysfunction, often present in combination with other NPSLE manifestations. The treatments mostly consisted of (combinations of) prednisone, methylprednisolone, azathioprine, and cyclophosphamide. The study recommended glucocorticoids alone or in combination with other immunosuppressants (e.g., azathioprine or cyclophosphamide) for the treatment of inflammatory NPSLE [153].

Glucocorticoids have inhibitory effects on a broad range of immune responses. They are often used to treat autoimmune diseases, including NPSLE. Glucocorticoids work by suppressing the immune system, which can help reduce inflammation and prevent damage to organs [154]. However, long-term use of glucocorticoids can lead to side effects such as weight gain, high blood pressure, and osteoporosis [153].

Glucocorticoids are the mainstay of lupus psychosis treatment. As NPSLE syndromes are suggested to be caused by autoimmune inflammatory processes, such as psychosis, an acute confusional state, and transverse myelitis, high-dose glucocorticoids and steroid-sparing agents such as cyclophosphamide and mycophenolate are the mainstay of treatment. Refractory cases of NPSLE can be treated using rituximab, intravenous immunoglobulin, or plasmapheresis [22].

Glucocorticoids are a class of steroid hormone that exerts its systemic and tissue-specific actions by binding glucocorticoid receptors. Glucocorticoids bind to the glucocorticoid receptor (GR) in the cytoplasm of target cells and modulate the expression of genes involved in inflammation, apoptosis, and metabolism [155]. They regulate the immune system by inhibiting the production of pro-inflammatory cytokines and chemokines, and by repressing the expression of pro-inflammatory cytokines by resident immune cells and extravasated immune cells. For instance, glucocorticoids have been shown to inhibit the production of cytokines such as interleukin-1 (IL-1), interleukin-6 (IL-6), and tumor necrosis factor-alpha (TNF-α) [156,157]. Glucocorticoids also inhibit the activation of macrophages and dendritic cells, and reduce the production of reactive oxygen species (ROS) and nitric oxide (NO). Glucocorticoids have been shown to interact with antibodies. They inhibit the production of immunoglobulin E (IgE) and immunoglobulin G (IgG), and reduce the expression of Fc receptors on immune cells. However, the molecular mechanisms underlying the systemic and tissue-specific actions of glucocorticoids are still a subject of intense investigation [157,158,159].

There are different types of glucocorticoids that vary in their potency, duration of action, and side effects. Some of the commonly used glucocorticoids in NPSLE are:

1. Prednisone is a synthetic glucocorticoid that is converted to its active form prednisolone in the liver. It has a moderate potency and a short half-life of about 3–4 h. Prednisone is usually given orally in doses ranging from 0.5 to 1 mg/kg/day for NPSLE [160];
2. Methylprednisolone is a synthetic glucocorticoid that has a higher potency and a longer half-life than prednisone of about 18–36 h. It can be given orally or intravenously in doses from 0.5 to 1 g/day for severe NPSLE [160];
3. Dexamethasone is a synthetic glucocorticoid that has a very high potency and a long half-life of about 36–54 h. It can be given orally or intravenously in doses from 10 to 100 mg/day for refractory NPSLE [160].

Glucocorticoids exert their anti-inflammatory and immunosuppressive effects by two main mechanisms:

Genomic mechanism: Glucocorticoids bind to the GR and form a complex that translocates to the nucleus and regulates the transcription of target genes. Some of these genes are upregulated by glucocorticoids and encode anti-inflammatory proteins, such as annexin A1, lipocortin, and IL-10. Other genes are downregulated by glucocorticoids and encode pro-inflammatory proteins, such as cytokines, chemokines, and adhesion molecules [155].

Non-genomic mechanism: Glucocorticoids can also bind to membrane-bound GRs and activate or inhibit various signaling pathways, such as MAPK, NF-κB, and PI3K/Akt. These pathways modulate the activity of inflammatory cells, such as macrophages, T cells, and B cells, and affect their survival, proliferation, differentiation, and function [155].

#### 7.2.2. Cyclophosphamide

Cyclophosphamide is a potent immunosuppressive agent that has been used in the treatment of neuropsychiatric systemic lupus erythematosus (NPSLE). It is often used for severe manifestations of NPSLE that are thought to reflect inflammation or an underlying autoimmune process [161]. Cyclophosphamide is the mainstay of NPSLE treatment. However, it is important to note that this drug can have serious side effects. For instance, a case report describes a patient with NPSLE who developed seizures soon after her first and second doses of low-dose cyclophosphamide. The exact pathophysiological mechanism underlying cyclophosphamide-induced seizures is not well- understood [161].

Despite its potential side effects, cyclophosphamide has been reported to benefit patients with systemic lupus erythematosus (SLE)-related Guillain-Barré syndrome, a rare neurological disorder [162]. These findings underscore the importance of careful monitoring and individualized treatment plans when using cyclophosphamide in the management of NPSLE [161,162].

Cyclophosphamide, as an immunosuppressive agent, works by suppressing the immune system and reducing the production of immune complexes, thereby reducing inflammation and damage to the nervous system. However, the use of cyclophosphamide can lead to serious side effects. For instance, severe acute hyponatraemic encephalopathy resulting from severe hyponatremia secondary to cyclophosphamide use has been reported [161].

#### 7.2.3. Azathioprine

Azathioprine is an immunosuppressive antimetabolite that has been used in the treatment of NPSLE. The mechanism of action of azathioprine is not entirely understood but it may be related to the inhibition of purine synthesis, along with the inhibition of B and T cells [163,164].

Azathioprine interferes with purine nucleic acid metabolism at steps required for lymphoid cell proliferation that follows antigenic stimulation. The purine analogs are cytotoxic and destroy stimulated lymphoid cells; 6-thioguanine triphosphate, a metabolite of azathioprine, modulates the activation of rac1 when co-stimulated with CD28, inducing T cell apoptosis [165]. This may be mediated through rac1′s action on the mitogen-activated protein kinase NF-kappaB [145].

Azathioprine’s effects as an antagonist of purine metabolism result in the inhibition of deoxyribonucleic acid (DNA), ribonucleic acid (RNA), and protein synthesis. This leads to a reduction in the immune response, which can help manage the symptoms of NPSLE [145,163].

#### 7.2.4. Mycophenolate Mofetil

Mycophenolate Mofetil (MMF) is an immunosuppressive drug that is often used in the treatment of various autoimmune diseases, including neuropsychiatric systemic lupus erythematosus (NPSLE) [39].

MMF is a prodrug of Mycophenolic Acid (MPA), which inhibits inosine monophosphate dehydrogenase (IMPDH), a key enzyme in the de novo synthesis pathway of guanosine nucleotides. This inhibition primarily affects lymphocytes, which rely heavily on this pathway for proliferation; thus, MMF has a selective effect on the immune system [166].

In NPSLE, MMF has been used as an alternative to cyclophosphamide for induction therapy in lupus nephritis, with comparable efficacy and a better safety profile [167]. A study comparing the efficacy of MMF with that of cyclophosphamide in lupus nephritis found that both drugs were effective, but that MMF had a better safety profile [168].

#### 7.2.5. Biologics

Rituximab, Belimumab, and Anifrolumab have been used in the treatment of NPSLE [39]. Rituximab is a monoclonal antibody that targets B cells, which play a key role in systemic lupus erythematosus (SLE). Clinical trials have shown that it can effectively deplete B cells and improve clinical outcomes [169,170]. Belimumab is an immunosuppressive drug that has been used as an alternative to cyclophosphamide for induction therapy in lupus nephritis, with comparable efficacy and a better safety profile [171]. Anifrolumab is a novel drug that targets the type I interferon pathway, which is implicated in the pathogenesis of SLE. Clinical trials have shown its potential benefits in treating these diseases [172].

### 7.3. Ischaemic Pathway

#### 7.3.1. Use of Aspirin in NPSLE

Aspirin is a nonsteroidal anti-inflammatory drug (NSAID) that has analgesic, antipyretic, anti-inflammatory, and antiplatelet effects [173]. Aspirin may be used in NPSLE for the following purposes:

- Prevention of thrombotic events: Aspirin inhibits the enzyme cyclooxygenase-1 (COX-1), which reduces the production of thromboxane A2, a prothrombotic mediator. This prevents platelet aggregation and reduces the risk of arterial and venous thrombosis, which can cause stroke, transient ischemic attack, or other neurological complications in NPSLE patients [174,175];
- Treatment of headache: Aspirin has analgesic and anti-inflammatory properties that can relieve headaches, one of the most common symptoms of NPSLE [176];
- Modulation of type I interferon response: Aspirin may have immunomodulatory effects on the type I interferon pathway, which is implicated in the pathogenesis of SLE and NPSLE. Aspirin may reduce the expression of interferon-stimulated genes and the levels of interferon-alpha, a cytokine that promotes inflammation and autoimmunity in NPSLE patients [177,178].

#### 7.3.2. Use of Heparin and Warfarin in NPSLE

Anticoagulant therapies, such as Heparin and Warfarin, can block the vicious cycle between inflammation and thrombosis, which may greatly improve the long-term prognosis of patients with SLE [179]. Heparin is often used as an initial treatment. It is given by injection and, in most cases, Warfarin (Coumadin), which is given orally, is then started. The level of anticoagulation is frequently monitored, most often using the INR test [180,181]. There is less evidence available on the use of Warfarin in patients with SLE. However, a prospective multicenter research trial showed that Warfarin (1–5 mg/day) started at the same time as a steroid therapy for at least 3 months can prevent the occurrence of osteonecrosis associated with SLE [179].

#### 7.3.3. Novel Oral Anticoagulants (NOACs)

Novel oral anticoagulants (NOACs) are a class of drugs that acts as direct inhibitors of either thrombin or factor Xa, two key enzymes in the coagulation cascade. They are used to prevent and treat thromboembolic disorders, such as stroke, deep vein thrombosis, and pulmonary embolism. Some of the NOACs that have been approved [182] are:

1. Dabigatran etexilate: a direct thrombin inhibitor that is approved for stroke prevention in atrial fibrillation, the prevention and treatment of venous thromboembolism, and the prevention of thromboembolism after hip or knee replacement surgery [183];
2. Rivaroxaban: a direct factor Xa inhibitor that is approved for stroke prevention in atrial fibrillation, the prevention and treatment of venous thromboembolism, the prevention of thromboembolism after hip or knee replacement surgery, and the secondary prevention of acute coronary syndrome [182];
3. Apixaban: a direct factor Xa inhibitor that is approved for stroke prevention in atrial fibrillation, the prevention and treatment of venous thromboembolism, and the prevention of thromboembolism after hip or knee replacement surgery [184];
4. Edoxaban: a direct factor Xa inhibitor that is approved for stroke prevention in atrial fibrillation and the treatment of venous thromboembolism [185].

NPSLE syndromes attributed to the prothrombotic state due to the presence of antiphospholipid antibodies warrant the use of anticoagulants and antiplatelet drugs. Anticoagulation may be superior to antiplatelet therapy for the secondary prevention of thrombotic events in patients undergoing antiphospholipid therapy [22].

### 7.4. Other Treatments

#### 7.4.1. Intravenous Immunoglobulins (IVIGs)

IVIGs have been used with success in autoimmune conditions, e.g., Kawasaki’s disease and idiopathic thrombotic purpura. IVIGs are an experimental form of the management of SLE. IVIGs are beneficial in autoimmune conditions due to their immunomodulatory effects via Fc receptor blockage, complement regulation, T cell regulation, and anti-idiotype regulation [186]. IVIGs have been shown to decrease serum titers of anti-dsDNA, reduce proteinuria, and decrease daily steroid requirements, which can be beneficial in patients with lupus nephritis [187]. IVIGs have also demonstrated benefits in the management of SLE during the maintenance phase, in cases of lupus flares, and in refractory cases [188]. While more definitive research is required, IVIGs appear to reduce disease activity in SLE patients along with improving complement levels [187].

#### 7.4.2. Non-Pharmacological Intervention in NPSLE

Recent studies have shed light on the role of certain proteins, such as S100A8/A9, in the immunopathogenesis of NPSLE. S100A8/A9 is a marker of inflammation and has been found to have higher concentrations in the serum of NPSLE patients compared to non-NPSLE patients. This protein has been linked to various neurological diseases and may play a role in the development of NPSLE [189].

While pharmacological interventions, including immunosuppression and psychiatric therapy, are imperative for managing NPSLE [18,39,137,145], non-pharmacological interventions also play a crucial role. Non-pharmacological interventions can help manage symptoms, improve patients’ quality of life, and potentially influence the course of the disease. However, specific non-pharmacological interventions for NPSLE are not well-documented in the literature. In general, non-pharmacological interventions for autoimmune diseases like SLE often involve lifestyle modifications, such as regular exercise, a balanced diet, adequate sleep, and stress-management techniques.

Exercise: Exercise stimulates cellular immunity by increasing the circulation of immune cells in the individual. This helps the body better prepare for a future infection by detecting it earlier. A study conducted in rats showed that muscle inflammation caused by exertion mobilizes inflammation-countering T cells, or Tregs, which enhance the muscles’ ability to use energy as fuel and improve overall exercise endurance [190,191]. Physical exercise may have beneficial effects on the Health-Related Quality of Life (HRQoL), disease activity, fatigue, depression, pain, and inflammatory markers in SLE patients, as well as preventing cardiovascular complications. It includes aerobic, resistance, and flexibility exercises [145,148].

Balanced Diet: Nutrients obtained through a balanced diet are essential for growth, cellular function, tissue development, energy, and immune defense. Macronutrients (proteins, carbohydrates, fatty acids) and micronutrients (vitamins, minerals, phytochemicals, antioxidants, probiotics) play an important role in modulating immune homeostasis. Nutrients exert their role in innate immunity and inflammation by regulating the expression of TLRs, as well as pro- and anti-inflammatory cytokines, thus interfering with immune cell crosstalk and signaling [192,193].

Adequate Sleep: Sleep has long been linked to immune function. A study showed that getting enough sleep influenced the environment where monocytes—a type of white blood cell-form—develop and get primed to support immune function. This process, hematopoiesis, occurs in the bone marrow [194,195].

Stress Management Techniques: The so-called “fight-or-flight” response heightens immune responsiveness. This response is associated with PBMC (peripheral blood mononuclear cells) expression profiles related to immune defense and recovery in swine [196].

Psychoeducational interventions: These are group-based programs that aim to improve memory, self-efficacy, coping skills, and the quality of life in SLE patients with cognitive dysfunction or mild NP symptoms. They may also reduce depression, anxiety, and fatigue. These interventions combine the elements of cognitive behavior therapy, group therapy, and education to provide patients and their families with knowledge about various facets of the illness and its treatment so that they can work together with mental health professionals for a better overall outcome. A meta-analysis of studies evaluating the effectiveness of passive psychoeducational interventions in reducing depression, anxiety, or psychological distress compared to no intervention, attention-placebo, or waitlist comparison groups revealed that brief passive psychoeducational interventions for depression and psychological distress can reduce symptoms. The quality of psychoeducation may be important in achieving positive outcomes [197].

Complementary and alternative medicine (CAM): This is a broad category that encompasses various modalities such as acupuncture, massage, herbal medicine, yoga, and meditation. CAM may offer some relief for NP symptoms such as headache, mood disorder, and anxiety, as well as improving the HRQoL [148]. Complementary and alternative medicine (CAM) has been found to be effective in improving immune function in patients with SLE. Innate immune cells and molecules have been found to play a key role in promoting and potentiating SLE. Recent studies have highlighted the involvement of different innate immune cells and pathways in the pathogenesis of SLE. The overproduction of cytokines such as interferons and interleukins causes the immune system to become overactive, leading to increased inflammation and tissue injury [198,199]. 

Laser treatment/phototherapy: This technique uses light energy to stimulate tissue healing, reduce inflammation, and modulate pain. Laser treatment/phototherapy may be useful for NPSLE patients with skin lesions and oral ulcers [200]. Collagen is a protein that is essential for wound healing and tissue repair. It is a fibrillar protein that constitutes a major component of the conjunctive and connective tissues, providing mechanical stability, elasticity, and strength to organisms [201].

Other molecules that have been shown to improve healing and inflammation include hyaluronic acid, vitamin C, and curcumin. Hyaluronic acid is a natural component of the extracellular matrix and has been shown to promote wound healing by increasing cell migration and proliferation. Vitamin C is an antioxidant that is involved in collagen synthesis and has been shown to improve wound healing. Curcumin is a natural anti-inflammatory compound found in turmeric and has been shown to reduce inflammation and improve wound healing [202,203].

In summary, non-pharmacological interventions, in conjunction with pharmacological treatments, can play a vital role in managing NPSLE. They can help manage symptoms and improve patients’ quality of life. However, more research is needed to identify specific non-pharmacological interventions for NPSLE and to understand their impact on the immunopathogenesis of the disease. As our understanding of NPSLE continues to evolve, it is hoped that more effective interventions, both pharmacological and non-pharmacological, will be developed to improve the lives of those affected by this complex disorder [148].

These interventions may be linked to the immunopathogenesis of NPSLE by modulating the inflammatory, autoimmune, and vascular processes that underlie NPSLE. However, more studies are needed to confirm their efficacy and safety, as well as to identify the optimal type, dose, and duration of these interventions.

## 8. Conclusions

The immunopathogenesis of NPSLE is complex and remains a major cause of mortality in patients with SLE. The immune mediators of NPSLE include genes, cytokines, chemokines, cells, and autoantibodies. Despite the increasing number of biomarkers and autoantibodies that have been tested and advancements in imaging techniques, there is still not a “gold standard” for the diagnosis of NPSLE.

Treatment options often require multiple medical therapies to manage symptoms. Immunosuppressants are the mainstay of management, and the use of antidepressants, antipsychotics, anxiolytics, and antiseizure medications is often required. Biologics such as Rituxumab, Belimumab, and Anifrolumab; anticoagulants including Aspirin, Heparin, Warfarin; and novel oral anticoagulants, plasmapheresis, intravenous immunoglobulins, and non-pharmacological interventions all play a critical role in the management of this neuropsychiatric and autoimmune condition.

## Figures and Tables

**Table 1:** Clinical syndromes in NPSLE. Taken from [18].

Central Nervous System	Neurological syndromes (focal): Seizure disorder Aseptic meningitis Cerebrovascular disease Demyelinating syndromes Headache Myelopathy Movement disorders Neuropsychiatric syndrome (diffuse): Anxiety disorders Psychosis Mood disorders Acute confusional state Cognitive dysfunction
Peripheral Central Nervous System	Neurological syndromes (focal): Autonomic disorders Myasthenia gravis Polyneuropathy Guillian Barre Syndrome Plexopathy Mononeuropathy

**Table 2:** Illustrating common NPSLE-associated symptoms in NPSLE.

Autoantibody	Location Isolated	Associated NPSLE Symptoms	References
Phospholipid: β2-glycoprotein 1 and cardiolipin (aCL-Ab)	Serum, CSF	CVD, seizures, chorea cognitive dysfunction, psychosis, depression, headache	[54,55,56,57,58]
Ribosomal P protein (anti-ribosmal P Ab)	Serum, CSF	psychosis, depression, cognitive impairment	[66,67,68]
NMDA receptor (anti-NMDA)	Serum, CSF	depression cognitive dysfunction	[84]
MAP-2 (anti-MAP-2 Ab)	Serum, CSF	seizures, chorea, sensory neuropathy, psychosis, headache)	[90,91,92]
U1 ribonucleoprotein (Anti-U1RNP Ab)	Serum, CSF	Diffuse NPSLE symptoms	[98]
Structural endothelial proteins (AECA)	Serum	Psychosis, depression	[102,103]
Triosephosphate isomerase(anti-TPI Ab)	Serum, CSF	aseptic meningitis	[105]
GAPDH (anti-GAPDH Ab)	Serum	Involved in various in neurodegenerative disorders, increased intracranial pressure, cognitive dysfunction	[108,110,111]

## Data Availability

Data are contained within the article.
